# Nematodes from terrestrial and freshwater habitats in the Arctic

**DOI:** 10.3897/BDJ.2.e1165

**Published:** 2014-08-19

**Authors:** Oleksandr Holovachov

**Affiliations:** † Swedish Museum of Natural History, Department of Zoology, Stockholm, Sweden

**Keywords:** Alaska, Arctic, distribution, fauna, freshwater, Greenland, Iceland, Jan Mayen, Lena River, Northwest territories, Novaya Zemlya, Nunavut, Severnaya Zemlya, Svalbard, Taymyr, terrestrial.

## Abstract

We present an updated list of terrestrial and freshwater nematodes from all regions of the Arctic, for which records of properly identified nematode species are available: Svalbard, Jan Mayen, Iceland, Greenland, Nunavut, Northwest territories, Alaska, Lena River estuary, Taymyr and Severnaya Zemlya and Novaya Zemlya. The list includes 391 species belonging to 146 genera, 54 families and 10 orders of the phylum Nematoda.

## Introduction

Nematodes are one of the most numerous and abundant multicellular organisms on the planet in general and in the Arctic in particular. There are over 70 research papers published which include data on the fauna and distribution of nematodes in the region. Few faunistic overviews of the Arctic nematodes exist in the literature. The "Catalogue of free-living soil and fresh-water nematodes of Arctic and Subarctic" (Kuzmin and Gagarin 1990) includes a large number of subarctic nematodes and is normally inaccessible. Subsequent publication by Gagarin (2001b) focuses only on the fresh-water nematodes from the Russian Arctic and Subarctic, while Coulson and Refseth (2004) give a list of terrestrial and freshwater nematodes from Svalbard and Jan Mayen. The present paper aims to provide a comprehensive overview of all findings of nematodes in the entire Arctic region between the years of 1883 and 2013, with all species names adjusted according to the most recent classification and nomenclature.

## Materials and methods

**Figure 1.**
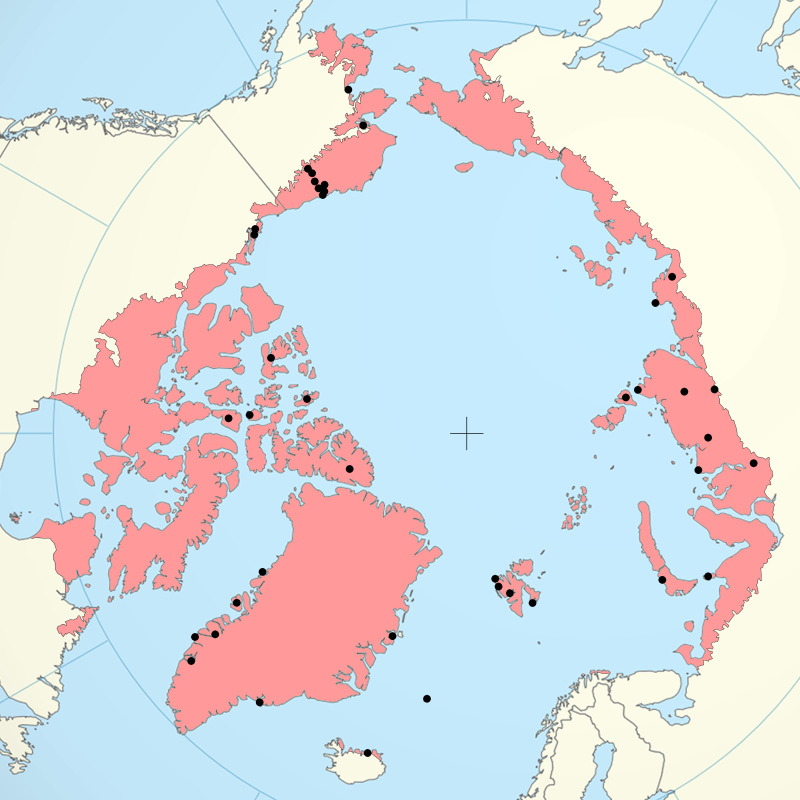
Known localities in the Arctic for which detailed information about nematode fauna (identified to species level) is available in the literature.

The species list has been compiled based on literature data and refers to the area of the Arctic (Fig. 1) as defined by the Circumpolar Arctic Vegetation Map (CAVM Team 2003) and Meltofte et al. (2013). The list below includes all species found in the Arctic, and specifies the regions where each species was found, as well as all relevant references. Current taxonomic status and systematic position of all species was updated according to the most recent views on nematode systematics. Section on nomenclature includes those synonymous names, and sometimes mistyped names, which were used in the original publications on the Arctic nematodes; it does not include any other existing synonyms or combinations. Asterisks indicate those new species names, which were originally proposed based on populations described from the Arctic.

Taxa identified in the literature to the genus level only were not considered in the review for the following reasons: 1) It is impossible to make sure how many actual species were considered by the author – just one, or if s/he had treated multiple species together in the same unit. 2) Taxa identified only to the genus level have limited usefulness when analyzing distribution, endemism and other aspects of biogeography. Nematode systematics is continuously improving. Menzel (1920) described *Dorylaimus* sp. from Spitzbergen (Svalbard). The context of the genus *Dorylaimus* had changed considerably since 1920 and now includes members of at least families Dorylaimidae, Qudsianematidae, Aporcelaimidae and Nordiidae, making it practically impossible to use Menzel's record in modern faunistic studies. Even genus-level identifications done more recently face similar problems. Species that are now considered to belong to the same genus may in future, when more data becomes available, be split into different, sometimes distantly related genera. It would thus be impossible to know in the future what author of the paper meant when he identified his material.

General classification and classification of the orders Chromadorida, Desmodorida and Araeolaimida follows De Ley and Blaxter (2004) and Holovachov and Esquivel (2012). Classification of the order Enoplida follows that of Smol et al. (2014) and Andrássy (2002); Triplonchida – Holovachov and Shoshin (2014); Dorylaimida – Andrássy (2009); Mononchida – Ahmad and Jairajpuri (2010); Monhysterida – Fonseca and Decraemer (2008); Plectida – Holovachov (2014); Rhabditida – Holovachov et al. (2009), Geraert (2008), Geraert (2011), Sudhaus (2011), Sudhaus and Fürst von Lieven (2003).

## Checklists

### Updated list of nematode species found in the Arctic

#### Nematoda

#### ENOPLIDA

#### Ironidae

#### Ironus tenuicaudatus

de Man, 1876

##### Notes

Taymyr and Severnaya Zemlya, Russia (Gagarin 1990, Gagarin 1993, Gagarin 1996, Gagarin 2001b, Gagarin 2001a, Kuzmin and Gagarin 1990).

#### Alaimidae

#### Alaimus arcuatus

Thorne, 1939

##### Notes

Svalbard (Loof 1971); Taymyr and Severnaya Zemlya, Russia (Kuzmin and Gagarin 1990).

#### Alaimus depressus

Loof, 1971*

##### Notes

Svalbard (Loof 1971).

#### Alaimus parvus

Thorne, 1939

##### Notes

Svalbard (Loof 1971).

#### Alaimus primitivus

de Man, 1880

##### Notes

Jan Mayen (Allgén 1953); Taymyr and Severnaya Zemlya, Russia (Gagarin 1990, Gagarin 2001b, Gagarin 2001a, Kuzmin and Gagarin 1990); Novaya Zemlya and Vaigach island, Russia (Steiner 1916b).

#### Alaimus proximus

Thorne, 1939

##### Notes

Taymyr and Severnaya Zemlya, Russia (Kuzmin 1986, Kuzmin and Gagarin 1990).

#### Alaimus similis

Thorne, 1939

##### Notes

Taymyr and Severnaya Zemlya, Russia (Kuzmin and Gagarin 1990).

#### Cosalaimus striatus

(Loof, 1964)

Alaimus striatus Loof, 1964

##### Notes

Taymyr and Severnaya Zemlya, Russia (Kuzmin 1986, Kuzmin and Gagarin 1990).

#### Amphidelus elegans

(de Man, 1921)

Alaimus elegans de Man, 1921

##### Notes

Jan Mayen (Allgén 1953); Taymyr and Severnaya Zemlya, Russia (Kuzmin and Gagarin 1990).

#### Paramphidelus dolichurus

(de Man, 1876)

Amphidelus dolichurus (de Man, 1876)

##### Notes

Svalbard (Loof 1971); Taymyr and Severnaya Zemlya, Russia (Kuzmin and Gagarin 1990).

#### Paramphidelus paludicola

Gagarin, 1991*

##### Notes

Taymyr and Severnaya Zemlya, Russia (Gagarin 1990, Gagarin 1991b, Gagarin 1993, Gagarin 2001b, Gagarin 2001a).

#### Paramphidelus paramonovi

(Eliashvili, 1971)

##### Notes

Novaya Zemlya and Vaigach island, Russia (Gagarin 1997a, Gagarin 2001b).

#### Paramphidelus pusillus

(Thorne, 1939)

Amphidelus pusillus Thorne, 1939

##### Notes

Taymyr and Severnaya Zemlya, Russia (Kuzmin and Gagarin 1990).

#### Rhabdolaimidae

#### Rhabdolaimus terrestris

de Man, 1880

##### Notes

Svalbard (Loof 1971); Nunavut, Canada (Mulvey 1969c); Taymyr and Severnaya Zemlya, Russia (Kuzmin 1976, Kuzmin and Gagarin 1990); Novaya Zemlya and Vaigach island, Russia (Gagarin 1997a, Gagarin 2001b).

#### Trefusiidae

#### Tripylina arenicola

(de Man, 1880)

Trischistoma arenicola (de Man, 1880)

##### Notes

Nunavut, Canada (Mulvey 1969c).

#### Trischistoma monohystera

(de Man, 1880)

##### Notes

Taymyr and Severnaya Zemlya, Russia (Kuzmin and Gagarin 1990).

#### TRIPLONCHIDA

#### Tobrilidae

#### Tobrilus aberrans

(W. Schneider, 1925)

##### Notes

Northwest territories, Canada (Ebsary 1982).

#### Tobrilus affinis

Gagarin, 1996*

##### Notes

Taymyr and Severnaya Zemlya, Russia (Gagarin 1996, Gagarin 2001b).

#### Tobrilus brevisetosus

(W. Schneider, 1925)

##### Notes

Taymyr and Severnaya Zemlya, Russia (Gagarin 2001b); Novaya Zemlya and Vaigach island, Russia (Gagarin 1997a, Gagarin 2001b).

#### Tobrilus gracilis

(Bastian, 1865)

##### Notes

Nunavut, Canada (Mulvey 1969c); Lena River estuary, Russia (Gagarin 2001b); Taymyr and Severnaya Zemlya, Russia (Gagarin 1990, Gagarin 1996, Gagarin 2001b, Gagarin 2001a, Gagarin and Kuzmin 1983, Kuzmin 1972, Kuzmin and Gagarin 1990); Novaya Zemlya and Vaigach island, Russia (Gagarin 1997a, Gagarin 1999a, Gagarin 2001b).

#### Tobrilus helveticus

(Hofmänner in Hofmänner & Menzel, 1914)

Trilobus pseudallophysis Micoletzky 1925

##### Notes

Greenland (Ditlevsen 1927); Taymyr and Severnaya Zemlya, Russia (Gagarin 1996, Kuzmin and Gagarin 1990).

#### Tobrilus modestus

Gagarin, 1996*

##### Notes

Taymyr and Severnaya Zemlya, Russia (Gagarin 1996, Gagarin 2001b).

#### Tobrilus parvus

Gagarin, 1991*

##### Notes

Taymyr and Severnaya Zemlya, Russia (Gagarin 1990, Gagarin 1991a, Gagarin 1993, Gagarin 2001b, Gagarin 2001a).

#### Tobrilus tripylis

Gagarin, 1991*

##### Notes

Taymyr and Severnaya Zemlya, Russia (Gagarin 1990, Gagarin 1991a, Gagarin 1993, Gagarin 2001b, Gagarin 2001a).

#### Brevitobrilus stefanskii

(Micoletzky, 1925)

##### Notes

Lena River estuary, Russia (Gagarin 2001b); Taymyr and Severnaya Zemlya, Russia (Gagarin 1990, Gagarin 1996, Gagarin 2001b, Gagarin 2001a, Kuzmin and Gagarin 1990); Novaya Zemlya and Vaigach island, Russia (Gagarin 1999a, Gagarin 2001b).

#### Epitobrilus allophysis

(Steiner, 1919)

##### Notes

Taymyr and Severnaya Zemlya, Russia (Gagarin 1996, Gagarin 2001b); Novaya Zemlya and Vaigach island, Russia (Gagarin 1997a, Gagarin 2001b).

#### Eutobrilus affectiosus

Shoshin, 1988

Eutobrilus antarcticus sensu Gagarin, 1993, 2001; *Raritobrilus antarcticus* sensu Gagarin 1990, 1991

##### Notes

Taymyr and Severnaya Zemlya, Russia (Gagarin 1990, Gagarin 1991a, Gagarin 1993, Gagarin 2001b, Gagarin 2001a, Gagarin 2009).

#### Eutobrilus altherri

(Andrássy, 1953)

##### Notes

Taymyr and Severnaya Zemlya, Russia (Gagarin 1996, Gagarin 2001b).

#### Eutobrilus andrassyi

(Altherr, 1963)

##### Notes

Taymyr and Severnaya Zemlya, Russia (Gagarin 2009, Kuzmin and Gagarin 1990).

#### Eutobrilus grandipapillatus

(Brakenhoff, 1914)

##### Notes

Lena River estuary, Russia (Gagarin 2001b); Taymyr and Severnaya Zemlya, Russia (Gagarin 1996, Gagarin 2001b); Novaya Zemlya and Vaigach island, Russia (Gagarin 1997a, Gagarin 1999a, Gagarin 2001b).

#### Eutobrilus selengaensis

(Tsalolikhin, 1977)

##### Notes

Taymyr and Severnaya Zemlya, Russia (Gagarin 1996, Gagarin 2001b).

#### Neotobrilus longus

(Leidy, 1852)

##### Notes

Lena River estuary, Russia (Gagarin 2001b); Taymyr and Severnaya Zemlya, Russia (Gagarin 1996, Kuzmin and Gagarin 1990).

#### Paratrilobus grandipapilloides

Micoletzky, 1922

##### Notes

Taymyr and Severnaya Zemlya, Russia (Gagarin 1990, Gagarin 1993, Gagarin 2001b, Gagarin 2001a).

#### Paratrilobus rapis

Gagarin, 1991*

##### Notes

Taymyr and Severnaya Zemlya, Russia (Gagarin 1990, Gagarin 1991a, Gagarin 1993, Gagarin 2001b, Gagarin 2001a).

#### Peritobrilus arcticus

(Gagarin, 1991)

Eutobrilus arcticus Gagarin, 1991*

##### Notes

Taymyr and Severnaya Zemlya, Russia (Gagarin 1990, Gagarin 1991a, Gagarin 1993, Gagarin 2001b, Gagarin 2001a).

#### Peritobrilus vipriensis

Gagarin, 1999*

##### Notes

Lena River estuary, Russia (Gagarin 1999b, Gagarin 2001b).

#### Quasibrilus strenuus

(Gagarin, 1991)

Eutobrilus strenuus Gagarin, 1991*

##### Notes

Taymyr and Severnaya Zemlya, Russia (Gagarin 1990, Gagarin 1991a, Gagarin 1993, Gagarin 2001b, Gagarin 2001a).

#### Raritobrilus steineri

(Micoletzky, 1925)

Eutobrilus steineri (Micoletzky, 1925); *Tobrilus steineri* Micoletzky, 1925

##### Notes

Lena River estuary, Russia (Gagarin 2001b); Taymyr and Severnaya Zemlya, Russia (Gagarin 1990, Gagarin 1993, Gagarin 1996, Gagarin 2001b, Gagarin 2001a, Kuzmin 1986, Kuzmin and Gagarin 1990).

#### Semitobrilus ebsaryi

Tsalolikhin, 2000*

Tobrilus longicaudatus sensu Ebsary, 1982

##### Notes

Northwest territories, Canada (Ebsary 1982).

#### Semitobrilus longicaudatus

(Schneider, 1923)

Tobrilus longicaudatus (Schneider, 1923)

##### Notes

Taymyr and Severnaya Zemlya, Russia (Kuzmin 1972, Kuzmin and Gagarin 1990).

#### Semitobrilus parapellucidus

(Ebsary, 1982)

Tobrilus parapellucidus Ebsary, 1982*

##### Notes

Northwest territories, Canada (Ebsary 1982); Taymyr and Severnaya Zemlya, Russia (Gagarin 1990, Gagarin 1993, Gagarin 2001b, Gagarin 2001a).

#### Semitobrilus gagarini

(Ebsary, 1982)

##### Notes

Lena River estuary, Russia (Gagarin 2001b).

#### Tripylidae

#### Tripyla affinis

de Man, 1880

##### Notes

Nunavut, Canada (Mulvey 1969c); Taymyr and Severnaya Zemlya, Russia (Kuzmin and Gagarin 1990); Novaya Zemlya and Vaigach island, Russia (Steiner 1916b).

#### Tripyla dubia

Gagarin, 1997*

##### Notes

Novaya Zemlya and Vaigach island, Russia (Gagarin 1997a, Gagarin 2001b).

#### Tripyla filicaudata

de Man, 1880

##### Notes

Taymyr and Severnaya Zemlya, Russia (Kuzmin 1986, Kuzmin and Gagarin 1990).

#### Tripyla glomerans

Bastian, 1865

Tripyla papillata Bütschli, 1873

##### Notes

Svalbard (Loof 1971); Greenland (Ditlevsen 1927); Taymyr and Severnaya Zemlya, Russia (Gagarin 1996, Kuzmin 1972, Kuzmin and Gagarin 1990); Novaya Zemlya and Vaigach island, Russia (Gagarin 1997a, Gagarin 1999a, Gagarin 2001b).

#### Tripyla infia

Brzeski & Winiszewska-Slipinska, 1993

Tripyla filipjevi Altherr in Altherr & Delamare Deboutteville, 1972

##### Notes

Svalbard (Janiec 1997); Taymyr and Severnaya Zemlya, Russia (Gagarin 1990, Gagarin 1993, Gagarin 1996, Gagarin 2001b, Gagarin 2001a).

#### Tripyla magna

Altherr in Altherr & Delamare Deboutteville, 1972

##### Notes

Lena River estuary, Russia (Gagarin 2001b); Taymyr and Severnaya Zemlya, Russia (Gagarin 1990, Gagarin 1993, Gagarin 1996, Gagarin 2001b, Gagarin 2001a).

#### Tripyla setifera

Bütschli, 1873

##### Notes

Lena River estuary, Russia (Gagarin 2001b); Taymyr and Severnaya Zemlya, Russia (Kuzmin and Gagarin 1990).

#### Prismatolaimidae

#### Prismatolaimus dolichurus

de Man, 1880

##### Notes

Svalbard (Loof 1971); Jan Mayen (Allgén 1953); Greenland (Ditlevsen 1927); Taymyr and Severnaya Zemlya, Russia (Kuzmin and Gagarin 1990); Novaya Zemlya and Vaigach island, Russia (Steiner 1916b).

#### Prismatolaimus intermedius

(Bütschli, 1873)

##### Notes

Svalbard (Loof 1971); Jan Mayen (Allgén 1953); Nunavut, Canada (Mulvey 1969c); Taymyr and Severnaya Zemlya, Russia (Kuzmin and Gagarin 1990).

#### Prismatolaimus primitivus

Loof, 1971*

##### Notes

Svalbard (Loof 1971).

#### Prismatolaimus stenolaimoides

Loof, 1971*

##### Notes

Svalbard (Loof 1971).

#### Prismatolaimus tareya

Gagarin & Kuzmin, 1972*

##### Notes

Taymyr and Severnaya Zemlya, Russia (Gagarin 1993, Gagarin and Kuzmin 1972, Kuzmin 1976, Kuzmin and Gagarin 1990).

#### Prismatolaimus verrucosus

Hirschmann, 1952

##### Notes

Novaya Zemlya and Vaigach island, Russia (Gagarin 1997a, Gagarin 2001b).

#### Bastianiidae

#### Bastiania gracilis

de Man, 1876

##### Notes

Svalbard (Loof 1971); Nunavut, Canada (Mulvey 1969c); Taymyr and Severnaya Zemlya, Russia (Kuzmin and Gagarin 1990); Novaya Zemlya and Vaigach island, Russia (Steiner 1916b).

#### Bastiania parexilis

De Coninck, 1935

##### Notes

Taymyr and Severnaya Zemlya, Russia (Kuzmin and Gagarin 1990).

#### Odontolaimidae

#### Odontolaimus chlorurus

de Man, 1880

##### Notes

Svalbard (Loof 1971); Taymyr and Severnaya Zemlya, Russia (Kuzmin and Gagarin 1990).

#### Diphtherophoridae

#### Diphtherophora communis

de Man, 1880

##### Notes

Taymyr and Severnaya Zemlya, Russia (Kuzmin and Gagarin 1990).

#### Diphtherophora minuta

Ivanova, 1958

##### Notes

Taymyr and Severnaya Zemlya, Russia (Kuzmin and Gagarin 1990).

#### Diphtherophora obesa

Thorne, 1939

##### Notes

Taymyr and Severnaya Zemlya, Russia (Kuzmin and Gagarin 1990).

#### Diphtherophora perplexans

(Cobb, 1913)

##### Notes

Taymyr and Severnaya Zemlya, Russia (Kuzmin and Gagarin 1990).

#### Tylolaimophorus bulgaricus

(Andrássy, 1958)

##### Notes

Taymyr and Severnaya Zemlya, Russia (Kuzmin and Gagarin 1990).

#### Tylolaimophorus cylindricus

(Cobb, 1920)

##### Notes

Taymyr and Severnaya Zemlya, Russia (Kuzmin and Gagarin 1990).

#### Trichodoridae

#### Trichodorus californicus

Allen, 1957

##### Notes

Alaska (Bernard 1992).

#### Trichodorus carlingi

Bernard, 1992*

##### Notes

Alaska (Bernard 1992).

#### DORYLAIMIDA

#### Nygolaimidae

#### Paravulvus hartingii

(de Man, 1880)

##### Notes

Taymyr and Severnaya Zemlya, Russia (Gagarin 1990, Gagarin 1996, Gagarin 2001b, Gagarin 2001a).

#### Clavicaudoides clavicaudatus

(Altherr, 1953)

##### Notes

Taymyr and Severnaya Zemlya, Russia (Kuzmin and Gagarin 1990).

#### Solididens vulgaris

(Thorne, 1930)

##### Notes

Taymyr and Severnaya Zemlya, Russia (Kuzmin and Gagarin 1990).

#### Dorylaimidae

#### Calcaridorylaimus arcticus

Gagarin, 1997*

##### Notes

Novaya Zemlya and Vaigach island, Russia (Gagarin 1997a, Gagarin 2001b).

#### Calcaridorylaimus promissus

Andrássy, 1986

##### Notes

Alaska (Andrássy 2003c).

#### Chrysodorus filiformis

(Bastian, 1865)

Dorylaimus filiformis Bastian, 1865

##### Notes

Greenland (Allgén 1954).

#### Crocodorylaimus biserovi

Gagarin, 1996*

##### Notes

Taymyr and Severnaya Zemlya, Russia (Gagarin 2001b).

#### Crocodorylaimus flavomaculatus

(Linstow, 1876)

Mesodorylaimus flavomaculatus (Linstow, 1876)

##### Notes

Taymyr and Severnaya Zemlya, Russia (Kuzmin and Gagarin 1990).

#### Crocodorylaimus dadayi

(Thorne & Swanger, 1936)

Laimydorus dadayi (Thorne & Swanger, 1936)

##### Notes

Taymyr and Severnaya Zemlya, Russia (Kuzmin 1986).

#### Dorylaimus lineatus

Altherr in Altherr & Delamare-Deboutteville, 1972

##### Notes

Northwest territories, Canada (Mulvey and Anderson 1979).

#### Dorylaimus stagnalis

Dujardin, 1845

##### Notes

Greenland (Ditlevsen 1927); Northwest territories, Canada (Mulvey and Anderson 1979); Lena River estuary, Russia (Gagarin 2001b); Taymyr and Severnaya Zemlya, Russia (Gagarin 1990, Gagarin 1996, Gagarin 2001b, Gagarin 2001a, Kuzmin and Gagarin 1990); Novaya Zemlya and Vaigach island, Russia (Gagarin 1997a, Gagarin 2001b).

#### Laimydorus doryuris

(Ditlevsen, 1911)

##### Notes

Taymyr and Severnaya Zemlya, Russia (Kuzmin and Gagarin 1990).

#### Laimydorus finalis

Thorne, 1975

##### Notes

Novaya Zemlya and Vaigach island, Russia (Gagarin 1997a, Gagarin 2001b).

#### Laimydorus pseudostagnalis

(Micoletzly, 1927)

##### Notes

Taymyr and Severnaya Zemlya, Russia (Kuzmin and Gagarin 1990); Novaya Zemlya and Vaigach island, Russia (Gagarin 1997a, Gagarin 2001b).

#### Mesodorylaimus bastiani

(Bütschli, 1873)

Dorylaimus bastiani Bütschli, 1873; *Dorylaimus langii* Cobb, 1888

##### Notes

Svalbard (von Linstow 1900); Taymyr and Severnaya Zemlya, Russia (Kuzmin and Gagarin 1990); Novaya Zemlya and Vaigach island, Russia (Steiner 1916b).

#### Mesodorylaimus japonicus

(Cobb in Thorne & Swanger, 1936)

##### Notes

Taymyr and Severnaya Zemlya, Russia (Kuzmin and Gagarin 1990).

#### Mesodorylaimus litoralis

Loof, 1969

##### Notes

Taymyr and Severnaya Zemlya, Russia (Gagarin 1990, Gagarin 2001b, Gagarin 2001a).

#### Mesodorylaimus pendschikenticus

(Tulaganov, 1949)

##### Notes

Taymyr and Severnaya Zemlya, Russia (Gagarin 1990, Gagarin 2001b, Gagarin 2001a).

#### Actinolaimidae

#### Paractinolaimus macrolaimus

(de Man, 1884)

Actinolaimus macrolaimus (de Man, 1884)

##### Notes

Greenland (Ditlevsen 1927); Alaska (Andrássy 2003c); Lena River estuary, Russia (Gagarin 2001b); Taymyr and Severnaya Zemlya, Russia (Kuzmin 1972, Kuzmin and Gagarin 1990).

#### Paractinolaimus longidrilus

Eveleigh, 1982*

##### Notes

Northwest territories, Canada (Eveleigh 1982).

#### Westindicus cinctus

(Cobb in Thorne, 1939)

##### Notes

Taymyr and Severnaya Zemlya, Russia (Kuzmin and Gagarin 1990).

#### Qudsianematidae

#### Arctidorylaimus arcticus

Mulvey & Anderson, 1979*

##### Notes

Northwest territories, Canada (Mulvey and Anderson 1979).

#### Allodorylaimus andrassyi

(Meyl, 1955)

##### Notes

Taymyr and Severnaya Zemlya, Russia (Kuzmin and Gagarin 1990).

#### Allodorylaimus diadematus

(Cobb in Thorne & Swanger, 1936)

##### Notes

Taymyr and Severnaya Zemlya, Russia (Kuzmin and Gagarin 1990).

#### Allodorylaimus holdemani

(Andrássy, 1959)

##### Notes

Taymyr and Severnaya Zemlya, Russia (Kuzmin and Gagarin 1990).

#### Allodorylaimus lindbergi

(Andrássy, 1960)

Eudorylaimus lindbergi Andrássy, 1960; *Allodorylaimus rarus* Gagarin, 1999*; *Eudorylaimus curvicaudatus* Eliava, 1968

##### Notes

Lena River estuary, Russia (Gagarin 1999a, Gagarin 1999b, Gagarin 2001b); Taymyr and Severnaya Zemlya, Russia (Gagarin 1990, Gagarin 1996, Gagarin 1999a, Gagarin 2001a, Gagarin 2001b); Novaya Zemlya and Vaigach island, Russia (Gagarin 1997a, Gagarin 1999a, Gagarin 2001b).

#### Allodorylaimus uniformis

(Thorne, 1929)

##### Notes

Taymyr and Severnaya Zemlya, Russia (Kuzmin and Gagarin 1990); Novaya Zemlya and Vaigach island, Russia (Gagarin 1997a, Gagarin 1999a, Gagarin 2001b).

#### Eudorylaimus acuticauda

(de Man, 1880)

Dorylaimus acuticauda de Man, 1880; *Dorylaimus carteri acuticauda* Micoletzky, 1922; Dorylaimus carteri Bastian, 1865 sf. acuticaudata de Man, 1880 (lapsus); Dorylaimus carteri Bastian, 1865 var. brevicaudatus f. typica sf. acuticauda de Man, 1880

##### Notes

Jan Mayen (Allgén 1953); Greenland (Allgén 1954, Ditlevsen 1927); Taymyr and Severnaya Zemlya, Russia (Kuzmin 1972, Kuzmin and Gagarin 1990); Novaya Zemlya and Vaigach island, Russia (Steiner 1916b).

#### Eudorylaimus acutus

(Thorne & Swanger, 1936)

##### Notes

Taymyr and Severnaya Zemlya, Russia (Kuzmin 1986, Kuzmin and Gagarin 1990).

#### Eudorylaimus bureshi

(Andrássy, 1958)

##### Notes

Taymyr and Severnaya Zemlya, Russia (Kuzmin and Gagarin 1990).

#### Eudorylaimus carteri

(Bastian, 1865)

Dorylaimus carteri Bastian, 1865

##### Notes

Svalbard (Klekowski and Opalinski 1986, Klekowski and Opalinski 1990, Klekowski and Opalinski 1992, Opalinski and Klekowski 1992); Jan Mayen (Allgén 1953); Greenland (Ditlevsen 1927); Alaska (Andrássy 2003c); Taymyr and Severnaya Zemlya, Russia (Gagarin 1996, Gagarin and Kuzmin 1983, Kuzmin 1972, Kuzmin 1976, Kuzmin and Gagarin 1990); Novaya Zemlya and Vaigach island, Russia (Steiner 1916b).

#### Eudorylaimus centrocercus

(de Man, 1880)

Dorylaimus centrocercus de Man, 1880; *Aporcelaimellus centrocercus* (de Man, 1880)

##### Notes

Jan Mayen (Allgén 1953); Taymyr and Severnaya Zemlya, Russia (Gagarin 1990, Gagarin 2001b, Gagarin 2001a, Kuzmin and Gagarin 1990).

#### Eudorylaimus iners

(Bastian, 1865)

Dorylaimus gracilis de Man, 1876

##### Notes

Jan Mayen (Allgén 1953).

#### Eudorylaimus leuckarti

(Bütschli, 1873)

Dorylaimus carteri Bastian, 1865 var. brevicaudatus Micoletzly, 1922 f. typica Micoletzky, 1922

##### Notes

Greenland (Allgén 1954).

#### Eudorylaimus megodon

Loof, 1971*

##### Notes

Svalbard (Loof 1971).

#### Eudorylaimus subjunctus

Loof, 1971*

##### Notes

Svalbard (Loof 1971).

#### Eudorylaimus vanrosseni

Loof, 1971*

##### Notes

Svalbard (Loof 1971).

#### Epidorylaimus agilis

(de Man, 1880)

Dorylaimus agilis de Man, 1880; Dorylaimus carteri Bastian, 1865 var. agilis de Man, 1880; *Eudorylaimus agilis* (de Man, 1880); *Laimydorus agilis* (de Man, 1880)

##### Notes

Svalbard (Loof 1971); Jan Mayen (Allgén 1953); Greenland (Allgén 1954); Taymyr and Severnaya Zemlya, Russia (Kuzmin 1986, Kuzmin and Gagarin 1990); Novaya Zemlya and Vaigach island, Russia (Steiner 1916b).

#### Epidorylaimus angulosus

(Thorne & Swanger, 1936)

Eudorylaimus anquilosus (Thorne & Swanger, 1936)

##### Notes

Taymyr and Severnaya Zemlya, Russia (Kuzmin 1972, Kuzmin and Gagarin 1990).

#### Epidorylaimus consobrinus

(de Man, 1918)

##### Notes

Taymyr and Severnaya Zemlya, Russia (Gagarin 1996, Kuzmin and Gagarin 1990).

#### Epidorylaimus humilis

(Thorne & Swanger, 1936)

Eudorylaimus humilis (Thorne & Swanger, 1936)

##### Notes

Taymyr and Severnaya Zemlya, Russia (Kuzmin 1972, Kuzmin and Gagarin 1990).

#### Epidorylaimus lugdunensis

(de Man, 1880)

Dorylaimus carteri Bastian, 1865 sf. lugdunensis de Man, 1880; *Dorylaimus carteri lugdunensis* Micoletzky, 1922; *Eudorylaimus lugdunensis* (de Man, 1880); *Dorylaimus reisingeri* Ditlevsen, 1927*

##### Notes

Svalbard (Loof 1971); Jan Mayen (Allgén 1953); Greenland (Ditlevsen 1927); Alaska (Andrássy 2003c); Taymyr and Severnaya Zemlya, Russia (Kuzmin and Gagarin 1990).

#### Epidorylaimus muscorum

(Skwarra, 1921)

##### Notes

Taymyr and Severnaya Zemlya, Russia (Kuzmin and Gagarin 1990).

#### Epidorylaimus rivalis

Gagarin, 1991*

##### Notes

Taymyr and Severnaya Zemlya, Russia (Gagarin 1990, Gagarin 1991b, Gagarin 2001b, Gagarin 2001a).

#### Microdorylaimus parvus

(de Man, 1880)

Eudorylaimus parvus (de Man, 1880)

##### Notes

Svalbard (Loof 1971); Taymyr and Severnaya Zemlya, Russia (Kuzmin and Gagarin 1990).

#### Crassolabium circulifer

(Loof, 1961)

Eudorylaimus circulifer Loof, 1961

##### Notes

Svalbard (Loof 1971).

#### Crassolabium ettersbergense

(de Man, 1885)

Eudorylaimus ettersbergensis (de Man, 1885); *Thonus ettersbergensis* (de Man, 1885)

##### Notes

Taymyr and Severnaya Zemlya, Russia (Kuzmin 1972, Kuzmin and Gagarin 1990).

#### Crassolabium laticollis

(de Man, 1906)

Thonus laticollis (de Man, 1906)

##### Notes

Taymyr and Severnaya Zemlya, Russia (Kuzmin and Gagarin 1990).

#### Dorydorella bryophila

(de Man, 1880)

Dorylaimus carteri Bastian, 1865 sf. briophilus de Man, 1880

##### Notes

Jan Mayen (Allgén 1953).

#### Dorydorella pratensis

(de Man, 1880)

Dorylaimus carteri Bastian, 1865 sf. pratensis de Man, 1880

##### Notes

Jan Mayen (Allgén 1953).

#### Ecumenicus monohystera

(de Man, 1880)

##### Notes

Taymyr and Severnaya Zemlya, Russia (Kuzmin and Gagarin 1990).

#### Labronema fimbriatum

Thorne, 1939

##### Notes

Taymyr and Severnaya Zemlya, Russia (Kuzmin and Gagarin 1990).

#### Labronema goodeyi

Altherr in Altherr & Delamare-Deboutteville, 1972

##### Notes

Taymyr and Severnaya Zemlya, Russia (Gagarin 1990, Gagarin 2001b, Gagarin 2001a).

#### Labronema loeffleri

Andrássy, 1978

##### Notes

Lena River estuary, Russia (Gagarin 2001b); Novaya Zemlya and Vaigach island, Russia (Gagarin 1997a).

#### Labronema stechlinense

Altherr, 1968

##### Notes

Alaska (Andrássy 2003c).

#### Aporcelaimidae

#### Akrotonus striaticaudatus

(Cobb, 1906)

##### Notes

Taymyr and Severnaya Zemlya, Russia (Kuzmin and Gagarin 1990).

#### Aporcelaimellus krygeri

(Ditlevsen, 1928)

Eudorylaimus krygeri (Ditlevsen, 1928)

##### Notes

Taymyr and Severnaya Zemlya, Russia (Gagarin 2001b, Kuzmin 1972, Kuzmin and Gagarin 1990); Novaya Zemlya and Vaigach island, Russia (Gagarin 2001b).

#### Aporcelaimellus obscurus

(Thorne & Swanger, 1936)

Eudorylaimus obscurus (Thorne & Swanger, 1936)

##### Notes

Taymyr and Severnaya Zemlya, Russia (Kuzmin 1972, Kuzmin and Gagarin 1990).

#### Aporcelaimellus obtusicaudatus

(Bastian, 1865)

Dorylaimus obtusicaudatus Bastian, 1865; Dorylaimus obtusicaudatus Bastian, 1865 f. butschlii Micoletzky, 1922; *Aporcelaimus obtusicaudatus* (Bastian, 1865)

##### Notes

Jan Mayen (Allgén 1953, Allgén 1954); Greenland (Allgén 1954, Ditlevsen 1927); Taymyr and Severnaya Zemlya, Russia (Gagarin 1990, Gagarin 2001b, Gagarin 2001a, Kuzmin and Gagarin 1990).

#### Aporcelaimellus papillatus

(Bastian, 1865)

Aporcelaimus papillatus (Bastian, 1865)

##### Notes

Taymyr and Severnaya Zemlya, Russia (Kuzmin and Gagarin 1990).

#### Aporcelaimellus paraobtusicaudatus

(Micoletzky, 1922)

##### Notes

Taymyr and Severnaya Zemlya, Russia (Kuzmin and Gagarin 1990).

#### Aporcelaimellus tritici

(Bastian, 1865)

Dorylaimus tritici Bastian, 1865

##### Notes

Jan Mayen (Allgén 1953); Greenland (Allgén 1954).

#### Aporcelaimus eurydoris

(Ditlevsen, 1911)

##### Notes

Taymyr and Severnaya Zemlya, Russia (Kuzmin and Gagarin 1990).

#### Aporcelaimus polaris

Andrássy, 2003*

##### Notes

Alaska (Andrássy 2003c).

#### Aporcelaimus superbus

(de Man, 1880)

##### Notes

Taymyr and Severnaya Zemlya, Russia (Kuzmin and Gagarin 1990).

#### Metaporcelaimus labiatus

(de Man, 1880)

Dorylaimus labiatus de Man, 1880

##### Notes

Jan Mayen (Allgén 1953).

#### Crateronematidae

#### Chrysonema maksymovi

(Altherr, 1963)

Eudorylaimus maksymovi (Altherr, 1963)

##### Notes

Svalbard (Klekowski and Opalinski 1986, Klekowski and Opalinski 1990, Loof 1971).

#### Nordiidae

#### Enchodelus analatus

(Ditlevsen, 1927)

Dorylaimus (Doryllium) analatus Ditlevsen, 1927*

##### Notes

Svalbard (Loof 1971); Greenland (Ditlevsen 1927).

#### Enchodelus groenlandicus

(Ditlevsen, 1927)

Dorylaimus (Doryllium) groenlandicus Ditlevsen 1927*

##### Notes

Greenland (Ditlevsen 1927); Taymyr and Severnaya Zemlya, Russia (Elshishka et al. 2012).

#### Enchodelus cf. macrodoroides

(Steiner, 1914)

##### Notes

Svalbard (Loof 1971).

#### Enchodelus macrodorus

(de Man, 1880)

Dorylaimus macrodorus de Man, 1880

##### Notes

Svalbard (Loof 1971); Jan Mayen (Allgén 1953); Lena River estuary, Russia (Gagarin 2001b); Taymyr and Severnaya Zemlya, Russia (Kuzmin and Gagarin 1990); Novaya Zemlya and Vaigach island, Russia (Gagarin 1997a, Gagarin 2001b, Steiner 1916a).

#### Enchodelus makarovae

Elshishka et al., 2012*

##### Notes

Taymyr and Severnaya Zemlya, Russia (Elshishka et al. 2012).

#### Enchodelus parateres

Baqri & Jairajpuri, 1974

##### Notes

Taymyr and Severnaya Zemlya, Russia (Kuzmin 1986, Kuzmin and Gagarin 1990).

#### Enchodelus parvus

Loof, 1971*

##### Notes

Svalbard (Loof 1971); Taymyr and Severnaya Zemlya, Russia (Kuzmin 1986, Kuzmin and Gagarin 1990).

#### Enchodelus teres

Thorne, 1939

##### Notes

Taymyr and Severnaya Zemlya, (Kuzmin and Gagarin 1990).

#### Heterodorus arquatus

(Thorne, 1939)

Enchodelus arquatus Thorne, 1939

##### Notes

Nunavut, Canada (Elshishka et al. 2013); Taymyr and Severnaya Zemlya, Russia (Elshishka et al. 2013, Kuzmin and Gagarin 1990).

#### Heterodorus conicaudatus

(Ditlevsen, 1927)

Dorylaimus conicaudatus Ditlevsen, 1927*; *Enchodelus conicaudatus* (Ditlevsen, 1927)

##### Notes

Svalbard (Loof 1971); Greenland (Ditlevsen 1927).

#### Heterodorus magnificus

Altherr, 1952

##### Notes

Taymyr and Severnaya Zemlya, Russia (Elshishka et al. 2013).

#### Pungentus longidens

(Thorne & Swanger, 1936)

##### Notes

Taymyr and Severnaya Zemlya, Russia (Kuzmin and Gagarin 1990).

#### Rhyssocolpus alleni

(Brzeski, 1962)

Eudorylaimus alleni Brzeski, 1962*

##### Notes

Svalbard (Brzeski 1962, Loof 1971).

#### Rhyssocolpus aquilonius

Andrássy, 2003*

##### Notes

Alaska (Andrássy 2003c).

#### Rhyssocolpus arcticus

Ebsary, 1984*

##### Notes

Nunavut, Canada (Ebsary 1984).

#### Longidorella magna

Loof, 1971*

##### Notes

Svalbard (Loof 1971); Alaska (Andrássy 2003c).

#### Longidorella parva

Thorne, 1939

##### Notes

Taymyr and Severnaya Zemlya, Russia (Kuzmin and Gagarin 1990).

#### Longidorella penetrans

(Thorne & Swanger, 1936)

##### Notes

Taymyr and Severnaya Zemlya, Russia (Kuzmin and Gagarin 1990).

#### Leptonchidae

#### Xiphinemella eversum

Heyns, 1969

##### Notes

Taymyr and Severnaya Zemlya, Russia (Kuzmin and Gagarin 1990).

#### Tylencholaimidae

#### Tylencholaimus crassus

Loof & Jairajpuri, 1968

##### Notes

Taymyr and Severnaya Zemlya, Russia (Kuzmin and Gagarin 1990).

#### Tylencholaimus mirabilis

(Bütschli, 1873)

##### Notes

Taymyr and Severnaya Zemlya, Russia (Kuzmin and Gagarin 1990).

#### Tylencholaimus proximus

Thorne, 1939

##### Notes

Svalbard (Loof 1971); Taymyr and Severnaya Zemlya, Russia (Kuzmin and Gagarin 1990).

#### Tylencholaimus stecki

Steiner, 1914

##### Notes

Taymyr and Severnaya Zemlya, Russia (Kuzmin and Gagarin 1990).

#### Tylencholaimus teres

Thorne, 1939

##### Notes

Svalbard (Loof 1971); Taymyr and Severnaya Zemlya, Russia (Kuzmin 1972, Kuzmin 1976, Kuzmin and Gagarin 1990).

#### Mydonomidae

#### Dorylaimoides arcuicaudatus

Baqri & Jairajpuri, 1969

##### Notes

Taymyr and Severnaya Zemlya, Russia (Kuzmin 1972, Kuzmin and Gagarin 1990).

#### Tylencholaimellidae

#### Tylencholaimellus coronatus

Thorne, 1939

##### Notes

Taymyr and Severnaya Zemlya, Russia (Kuzmin 1972, Kuzmin and Gagarin 1990).

#### MONONCHIDA

#### Mononchidae

#### Mononchus absconditus

(Tsalolikhin, 1974)

##### Notes

Lena River estuary, Russia (Gagarin 2001b); Taymyr and Severnaya Zemlya, Russia (Gagarin 1990, Gagarin 1993, Gagarin 2001b, Gagarin 2001a); Novaya Zemlya and Vaigach island, Russia (Gagarin 1997a, Gagarin 2001b).

#### Mononchus angarensis

Gagarin, 1984

##### Notes

Taymyr and Severnaya Zemlya, Russia (Gagarin 1996, Gagarin 2001b).

#### Mononchus aquaticus

Coetzee, 1968

##### Notes

Taymyr and Severnaya Zemlya, Russia (Gagarin 2001b).

#### Mononchus maduei

Schneider, 1925

##### Notes

Northwest territories, Canada (Mulvey 1978).

#### Mononchus niddensis

Skwarra, 1921

##### Notes

Nunavut, Canada (Mulvey 1967b, Mulvey 1978).

#### Mononchus superbus

Mulvey, 1978*

##### Notes

Northwest territories, Canada (Mulvey 1978).

#### Mononchus tajmiris

Gagarin, 1991*

##### Notes

Taymyr and Severnaya Zemlya, Russia (Gagarin 1990, Gagarin 1991b, Gagarin 1993, Gagarin 2001a, Gagarin 2001b).

#### Mononchus truncatus

Bastian, 1865

##### Notes

Northwest territories, Canada (Mulvey 1978); Taymyr and Severnaya Zemlya, Russia (Gagarin 1990, Gagarin 1996, Gagarin 2001b, Gagarin 2001a, Kuzmin and Gagarin 1990); Novaya Zemlya and Vaigach island, Russia (Gagarin 1997a, Gagarin 2001b).

#### Paramononchus arcticus

Mulvey, 1978*

##### Notes

Northwest territories, Canada (Mulvey 1978).

#### Prionchulus longus

(Thorne, 1929)

##### Notes

Nunavut, Canada (Mulvey 1967a, Mulvey 1978); Taymyr and Severnaya Zemlya, Russia (Kuzmin and Gagarin 1990).

#### Prionchulus major

Gagarin, 2001*

##### Notes

Novaya Zemlya and Vaigach island, Russia (Gagarin 2001c, Gagarin 2001b).

#### Prionchulus muscorum

(Dujardin, 1845)

##### Notes

Svalbard (Klekowski and Opalinski 1986, Klekowski and Opalinski 1990); Nunavut, Canada (Mulvey 1967a); Alaska (Andrássy 2003a); Taymyr and Severnaya Zemlya, Russia (Kuzmin and Gagarin 1990); Novaya Zemlya and Vaigach island, Russia (Gagarin 1997a, Gagarin 2001b).

#### Prionchulus punctatus

Cobb, 1917

##### Notes

Nunavut, Canada (Mulvey 1967a).

#### Prionchulus sedatus

Gagarin, 2000*

##### Notes

Lena River estuary, Russia (Gagarin 2000, Gagarin 2001b).

#### Prionchulus spectabilis

(Ditlevsen, 1911)

Mononchus spectabilis Ditlevsen, 1911

##### Notes

Greenland (Ditlevsen 1927).

#### Clarkus macropapillatus

(Mulvey, 1967)

##### Notes

Taymyr and Severnaya Zemlya, Russia (Kuzmin and Gagarin 1990).

#### Clarkus papillatus

(Bastina, 1865)

Mononchus papillatus Bastian, 1865

##### Notes

Jan Mayen (Allgén 1953); Greenland (Allgén 1954, Ditlevsen 1927); Taymyr and Severnaya Zemlya, Russia (Kuzmin and Gagarin 1990); Novaya Zemlya and Vaigach island, Russia (Steiner 1916b).

#### Coomansus fletcherensis

Mulvey, 1978*

##### Notes

Nunavut, Canada (Mulvey 1978).

#### Coomansus gerlachei

(de Man, 1904)

##### Notes

Northwest territories, Canada (Mulvey 1978).

#### Coomansus parvus

(de Man, 1880)

##### Notes

Alaska (Andrássy 2003a).

#### Parkellus zschokkei

(Menzel, 1913)

Iotonchus zschokkei (Menzel, 1913)

##### Notes

Taymyr and Severnaya Zemlya, Russia (Kuzmin 1972, Kuzmin and Gagarin 1990).

#### Mylonchulidae

#### Mylonchulus brachyuris

(Bütschli, 1873)

Mononchus brachyuris Bütschli, 1873

##### Notes

Greenland (Ditlevsen 1927); Alaska (Andrássy 2003a); Taymyr and Severnaya Zemlya, Russia (Kuzmin and Gagarin 1990); Novaya Zemlya and Vaigach island, Russia (Steiner 1916b).

#### Mylonchulus gigas

Gagarin, 1993

##### Notes

Novaya Zemlya and Vaigach island, Russia (Gagarin 1997a, Gagarin 2001b).

#### Mylonchulus incurvus

Cobb, 1917

##### Notes

Taymyr and Severnaya Zemlya, Russia (Gagarin 2001b).

#### Mylonchulus lacustris

(Cobb in Cobb, 1915)

##### Notes

Northwest territories, Canada (Mulvey 1978).

#### Anatonchidae

#### Miconchus exilis

(Cobb, 1917)

##### Notes

Taymyr and Severnaya Zemlya, Russia (Kuzmin and Gagarin 1990).

#### Iotonchidae

#### Jensenonchus amplus

Andrássy, 2003*

##### Notes

Alaska (Andrássy 2003a).

#### CHROMADORIDA

#### Chromadoridae

#### Chromadorita arctica

Gagarin, 1999*

Chromadorida arctica Gagarin, 1999 (lapsus)

##### Notes

Novaya Zemlya and Vaigach island, Russia (Gagarin 1999a, Gagarin 2001b).

#### Chromadorita leuckarti

(de Man, 1876)

##### Notes

Lena River estuary, Russia (Gagarin 2001b); Taymyr and Severnaya Zemlya, Russia (Gagarin 1996); Novaya Zemlya and Vaigach island, Russia (Gagarin 1997a, Gagarin 2001b).

#### Punctodora ratzemburgensis

(Linstow, 1876)

##### Notes

Novaya Zemlya and Vaigach island, Russia (Gagarin 1997a, Gagarin 2001b).

#### Achromadoridae

#### Achromadora micoletzkyi

(Stefanski, 1915)

Cyatholaimus micoletzkyi Steiner, 1916*; *Achromadora steineri* Mulvey, 1969

##### Notes

Taymyr and Severnaya Zemlya, Russia (Kuzmin 1976, Kuzmin and Gagarin 1990); Novaya Zemlya and Vaigach island, Russia (Steiner 1916b).

#### Achromadora ruricola

(de Man, 1880)

##### Notes

Nunavut, Canada (Mulvey 1969c); Taymyr and Severnaya Zemlya, Russia (Kuzmin 1972, Kuzmin and Gagarin 1990).

#### Achromadora cf. semiarmata

Altherr, 1952

##### Notes

Svalbard (Loof 1971).

#### Achromadora tenax

(de Man, 1876)

##### Notes

Svalbard (Janiec 1997, Loof 1971).

#### Achromadora terricola

(de Man, 1880)

Cyatholaimus ornatus Steiner, 1916*

##### Notes

Greenland (Ditlevsen 1927); Nunavut, Canada (Mulvey 1969c); Lena River estuary, Russia (Gagarin 2001b); Taymyr and Severnaya Zemlya, Russia (Kuzmin and Gagarin 1990); Novaya Zemlya and Vaigach island, Russia (Gagarin 1997a, Gagarin 2001b, Steiner 1916a).

#### Ethmolaimidae

#### Ethmolaimus pratensis

de Man, 1880

Ethmolaimus arcticus Steiner, 1916*

##### Notes

Nunavut, Canada (Mulvey 1969c); Lena River estuary, Russia (Gagarin 2001b); Taymyr and Severnaya Zemlya, Russia (Gagarin 1990, Gagarin 1996, Gagarin 2001b, Gagarin 2001a, Kuzmin 1972, Kuzmin and Gagarin 1990); Novaya Zemlya and Vaigach island, Russia (Gagarin 1997a, Gagarin 2001b, Steiner 1916a).

#### DESMODORIDA

#### Microlaimidae

#### Microlaimus globiceps

de Man, 1880

##### Notes

Taymyr and Severnaya Zemlya, Russia (Kuzmin and Gagarin 1990).

#### Prodesmodora arctica

(Mulvey, 1969)

Microlaimus arcticus Mulvey 1969*

##### Notes

Nunavut, Canada (Mulvey 1969c); Novaya Zemlya and Vaigach island, Russia (Gagarin 1997a, Gagarin 2001b).

#### Prodesmodora circulata

(Micoletzky, 1913)

##### Notes

Lena River estuary, Russia (Gagarin 2001b); Taymyr and Severnaya Zemlya, Russia (Gagarin 1996, Kuzmin 1976, Kuzmin and Gagarin 1990); Novaya Zemlya and Vaigach island, Russia (Gagarin 1997a, Gagarin 2001b).

#### MONHYSTERIDA

#### Xyalidae

#### Hofmaenneria hazanensis

Mulvey, 1969*

##### Notes

Nunavut, Canada (Mulvey 1969c).

#### Daptonema dubium

(Bütschli, 1873)

##### Notes

Taymyr and Severnaya Zemlya, Russia (Gagarin 1996, Gagarin and Kuzmin 1983, Kuzmin and Gagarin 1990).

#### Daptonema fortis

Gagarin, 1993*

##### Notes

Taymyr and Severnaya Zemlya, Russia (Gagarin 1990, Gagarin 1993, Gagarin 2001b, Gagarin 2001a).

#### Daptonema sibiricum

Gagarin, 2000*

##### Notes

Lena River estuary, Russia (Gagarin 2000, Gagarin 2001b).

#### Daptonema tenuispiculum

(Ditlevsen, 1918)

##### Notes

Novaya Zemlya and Vaigach island, Russia (Gagarin 1999a, Gagarin 2001b).

#### Theristus agilis

(de Man, 1880)

Monhystera agilis de Man, 1880

##### Notes

Jan Mayen (Allgén 1953, Tchesunov and Pletnikova 1986); Taymyr and Severnaya Zemlya, Russia (Kuzmin 1972, Kuzmin 1976, Kuzmin and Gagarin 1990).

#### Theristus flevensis

Schuurmans Stekhoven, 1935

##### Notes

Novaya Zemlya and Vaigach island, Russia (Gagarin 1997b, Gagarin 2001a).

#### Monhysteridae

#### Eumonhystera barbata

Andrássy, 1981

##### Notes

Novaya Zemlya and Vaigach island, Russia (Gagarin 1997b, Gagarin 2001a).

#### Eumonhystera dispar

(Bastian, 1865)

Monhystera dispar Bastian, 1865

##### Notes

Svalbard (Loof 1971); Nunavut, Canada (Mulvey 1969c); Taymyr and Severnaya Zemlya, Russia (Gagarin 1996); Novaya Zemlya and Vaigach island, Russia (Gagarin 1997b, Gagarin 1997a, Gagarin 1999a, Gagarin 2001b).

#### Eumonhystera filiformis

(Bastian, 1865)

Monohystera filiformis Bastian, 1865 (lapsus); *Monhystera filiformis* Butschlii, 1873 (lapsus)

##### Notes

Svalbard (Janiec 1997, Loof 1971); Lena River estuary, Russia (Gagarin 2001b); Taymyr and Severnaya Zemlya, Russia (Kuzmin 1972, Kuzmin and Gagarin 1990); Novaya Zemlya and Vaigach island, Russia (Gagarin 1997a, Gagarin 2001b, Steiner 1916a).

#### Eumonhystera kuzmini

Gagarin, 1997*

##### Notes

Novaya Zemlya and Vaigach island, Russia (Gagarin 1997b, Gagarin 1997a, Gagarin 1999a, Gagarin 2001b).

#### Eumonhystera maxima

Gagarin, 1996

##### Notes

Taymyr and Severnaya Zemlya, Russia (Gagarin 2001b).

#### Eumonhystera papuana

(Daday, 1899)

##### Notes

Taymyr and Severnaya Zemlya, Russia (Gagarin 1996, Gagarin 2001b).

#### Eumonhystera pseudobulbosa

(Daday, 1896)

Monohystera pseudobulbosa Daday, 1896 (lapsus)

##### Notes

Greenland (Ditlevsen 1927).

#### Eumonhystera similis

(Bütschli, 1873)

##### Notes

Taymyr and Severnaya Zemlya, Russia (Gagarin 2001b, Kuzmin and Gagarin 1990).

#### Eumonhystera simplex

(de Man, 1880)

Monhystera simplex de Man, 1880

##### Notes

Taymyr and Severnaya Zemlya, Russia (Kuzmin 1972, Kuzmin and Gagarin 1990).

#### Eumonhystera tuporis

Gagarin, 1991

##### Notes

Novaya Zemlya and Vaigach island, Russia (Gagarin 1999a, Gagarin 2001b).

#### Eumonhystera vulgaris

(de Man, 1880)

Monhystera vulgaris de Man, 1880; *Monohystera vulgaris* de Man, 1880 (lapsus)

##### Notes

Svalbard (Janiec 1997, Loof 1971); Jan Mayen (Allgén 1953); Nunavut, Canada (Mulvey 1969c); Lena River estuary, Russia (Gagarin 2001b); Taymyr and Severnaya Zemlya, Russia (Gagarin 1996, Kuzmin and Gagarin 1990); Novaya Zemlya and Vaigach island, Russia (Gagarin 1997a, Gagarin 1999a, Gagarin 2001b, Steiner 1916a).

#### Geomonhystera aenariensis

(Meyl, 1953)

##### Notes

Novaya Zemlya and Vaigach island, Russia (Gagarin 1997a, Gagarin 2001b).

#### Geomonhystera paravillosa

(Meyl, 1954)

Monhystera paravillosa Meyl, 1954

##### Notes

Nunavut, Canada (Mulvey 1969c).

#### Geomonhystera villosa

(Butschli, 1873)

Monohystera villosa Butschli, 1873 (lapsus)

##### Notes

Svalbard (Loof 1971); Taymyr and Severnaya Zemlya, Russia (Kuzmin and Gagarin 1990); Novaya Zemlya and Vaigach island, Russia (Steiner 1916b).

#### Halomonhystera disjuncta

(Bastian, 1865)

Monhystera disjuncta Bastian, 1865

##### Notes

Jan Mayen (Allgén 1953); Novaya Zemlya and Vaigach island, Russia (Gagarin 1999a, Gagarin 2001b).

#### Monhystera amabilis

Gagarin, 1997*

##### Notes

Novaya Zemlya and Vaigach island, Russia (Gagarin 1997b, Gagarin 1997a, Gagarin 2001c, Gagarin 2001b).

#### Monhystera stagnalis

Bastian, 1865

##### Notes

Svalbard (Janiec 1997, Loof 1971); Taymyr and Severnaya Zemlya, Russia (Gagarin and Kuzmin 1983, Kuzmin and Gagarin 1990).

#### Monhystrella macrura

(de Man, 1880)

Monhystera macrura de Man, 1880

##### Notes

Jan Mayen (Allgén 1953).

#### Tridentula bidenticulata

(Gagarin, 1997)

Eumonhystera bidenticulata Gagarin, 1997*; *Tridentulus diplodenticulata* Gagarin, 1997 (lapsus); *Eumonhystera diplodenticulata* Gagarin, 1997 (lapsus)

##### Notes

Novaya Zemlya and Vaigach island, Russia (Gagarin 1997b, Gagarin 1997a, Gagarin 2001b).

#### Tridentula obscura

(Gagarin, 2000)

Tridentulus obscurus Gagarin, 2000

##### Notes

Novaya Zemlya and Vaigach island, Russia (Gagarin 2001b).

#### ARAEOLAIMIDA

#### Diplopeltidae

#### Cylindrolaimus baradlanus

Andrássy, 1959

##### Notes

Taymyr and Severnaya Zemlya, Russia (Kuzmin 1972, Kuzmin and Gagarin 1990).

#### Cylindrolaimus communis

de Man, 1880

##### Notes

Taymyr and Severnaya Zemlya, Russia (Kuzmin and Gagarin 1990).

#### Cylindrolaimus melancholicus

de Man, 1880

##### Notes

Svalbard (Loof 1971); Nunavut, Canada (Mulvey 1969c); Lena River estuary, Russia (Gagarin 2001b); Taymyr and Severnaya Zemlya, Russia (Kuzmin 1972, Kuzmin and Gagarin 1990).

#### PLECTIDA

#### Camacolaimidae

#### Deontolaimus papillatus

de Man, 1880

##### Notes

Greenland (Ditlevsen 1927).

#### Chronogastridae

#### Chronogaster typica

(de Man, 1921)

##### Notes

Taymyr and Severnaya Zemlya, Russia (Kuzmin and Gagarin 1990).

#### Plectidae

#### Anaplectus grandepapillatus

(Ditlevsen, 1928)

Anaplectus submersus (Hirschmann, 1952)

##### Notes

Svalbard (Janiec 1997); Alaska (Andrássy 2003b); Lena River estuary, Russia (Gagarin 2001b); Taymyr and Severnaya Zemlya, Russia (Gagarin 1990, Gagarin 1993, Gagarin 2001b, Gagarin 2001a, Kuzmin and Gagarin 1990).

#### Anaplectus granulosus

(Bastian, 1865)

Plectus granulosus Bastian, 1865

##### Notes

Svalbard (Loof 1971); Jan Mayen (Allgén 1953); Greenland (Ditlevsen 1927); Taymyr and Severnaya Zemlya, Russia (Gagarin 1990, Gagarin 1996, Gagarin 2001b, Gagarin 2001a, Kuzmin and Gagarin 1990); Novaya Zemlya and Vaigach island, Russia (Gagarin 1997a, Gagarin 1999a, Gagarin 2001b, Steiner 1916a).

#### Anaplectus porosus

Allen and Noffsinger, 1968

##### Notes

Svalbard (Klekowski and Opalinski 1986, Klekowski and Opalinski 1990, Klekowski and Opalinski 1992, Loof 1971).

#### Arctiplectus alaskanus

Andrássy, 2003*

##### Notes

Alaska (Andrássy 2003b).

#### Perioplectus labiosus

(Sanwal, 1968)

Periplectus labiosus Sanwal, 1968*

##### Notes

Nunavut, Canada (Sanwal 1968, Zell 1993).

#### Perioplectus secundus

Andrássy, 2003*

##### Notes

Alaska (Andrássy 2003b).

#### Plectus acuminatus

Bastian, 1865

##### Notes

Svalbard (Loof 1971); Alaska (Andrássy 2003b); Taymyr and Severnaya Zemlya, Russia (Gagarin 2001b, Kuzmin and Gagarin 1990); Novaya Zemlya and Vaigach island, Russia (Gagarin 1997a, Gagarin 2001b).

#### Plectus amorphotelus

Ebsary, 1985

##### Notes

Svalbard (Zell 1993).

#### Plectus aquatilis

Andrássy, 1984

##### Notes

Svalbard (Janiec 1997).

#### Plectus cf. armatus

Butschli, 1873

##### Notes

Svalbard (Loof 1971).

#### Plectus assimilis

Bütschli, 1873

Ceratoplectus assimilis (Bütschli, 1873)

##### Notes

Svalbard (Loof 1971); Greenland (Zell 1993); Alaska (Andrássy 2003b); Taymyr and Severnaya Zemlya, Russia (Kuzmin and Gagarin 1990).

#### Plectus cancellatus

Zullini, 1978

Plectus thornei sensu Zell, 1993

##### Notes

Nunavut, Canada (Ebsary 1985, Zell 1993).

#### Plectus cirratus

Bastian, 1865

##### Notes

Svalbard (Klekowski and Opalinski 1986, Klekowski and Opalinski 1990, Klekowski and Opalinski 1992, Menzel 1920); Jan Mayen (Allgén 1953); Greenland (Ditlevsen 1927); Alaska (Andrássy 2003b); Lena River estuary, Russia (Gagarin 2001b); Taymyr and Severnaya Zemlya, Russia (Gagarin 1990, Gagarin 1996, Gagarin 2001b, Gagarin 2001a, Kuzmin and Gagarin 1990); Novaya Zemlya and Vaigach island, Russia (Gagarin 1997a, Gagarin 1999a, Gagarin 2001b).

#### Plectus communis

Bütschli, 1873

##### Notes

Svalbard (Menzel 1920, Zell 1993); Iceland (Zell 1993); Greenland (Allgén 1954).

#### Plectus cornus

Maggenti, 1961

Ceratoplectus cornus (Maggenti, 1961)

##### Notes

Svalbard (Loof 1971, Zell 1993); Taymyr and Severnaya Zemlya, Russia (Kuzmin 1976, Kuzmin and Gagarin 1990).

#### Plectus elongatus

Maggenti, 1961

##### Notes

Taymyr and Severnaya Zemlya, Russia (Kuzmin and Gagarin 1990).

#### Plectus geophilus

de Man, 1880

Plectus minor Novikova & Gagarin, 1971

##### Notes

Svalbard (Loof 1971, Zell 1993); Jan Mayen (Allgén 1953); Taymyr and Severnaya Zemlya, Russia (Kuzmin 1986, Kuzmin and Gagarin 1990).

#### Plectus inquirendus

Andrássy, 1958

##### Notes

Svalbard (Loof 1971, Zell 1993); Alaska (Andrássy 2003b); Taymyr and Severnaya Zemlya, Russia (Kuzmin and Gagarin 1990).

#### Plectus intermedius

Cobb, 1893

##### Notes

Nunavut, Canada (Zell 1993).

#### Plectus longicaudatus

Butschli, 1873

##### Notes

Svalbard (Loof 1971, Zell 1993); Jan Mayen (Allgén 1953); Iceland (Zell 1993); Taymyr and Severnaya Zemlya, Russia (Kuzmin and Gagarin 1990); Novaya Zemlya and Vaigach island, Russia (Steiner 1916b).

#### Plectus magadani

Kuzmin, 1979

##### Notes

Svalbard (Zell 1993).

#### Plectus murrayi

Yeates, 1970

##### Notes

Svalbard (Zell 1993).

#### Plectus cf. opisthocirculus

Andrássy, 1952

##### Notes

Svalbard (Janiec 1997).

#### Plectus palustris

de Man, 1880

##### Notes

Lena River estuary, Russia (Gagarin 2001b); Taymyr and Severnaya Zemlya, Russia (Gagarin 1996, Gagarin 2001b); Novaya Zemlya and Vaigach island, Russia (Gagarin 1997a, Gagarin 1999a, Gagarin 2001b).

#### Plectus parietinus

Bastian, 1865

Plectus cirratus Bastian, 1865 f. parietinus Bastian, 1865

##### Notes

Svalbard (Janiec 1997, Klekowski and Opalinski 1986, Klekowski and Opalinski 1990, Klekowski and Opalinski 1992, Loof 1971, Opalinski and Klekowski 1992); Jan Mayen (Allgén 1953); Iceland (Zell 1993); Alaska (Andrássy 2003b); Taymyr and Severnaya Zemlya, Russia (Gagarin 1990, Gagarin 1996, Gagarin 2001b, Gagarin 2001a, Gagarin and Kuzmin 1983, Kuzmin 1976, Kuzmin and Gagarin 1990); Novaya Zemlya and Vaigach island, Russia (Gagarin 1997a, Gagarin 2001b, Steiner 1916a).

#### Plectus parvus

Bastian, 1865

##### Notes

Svalbard (Loof 1971, Zell 1993); Iceland (Zell 1993); Taymyr and Severnaya Zemlya, Russia (Kuzmin and Gagarin 1990); Novaya Zemlya and Vaigach island, Russia (Gagarin 1997a, Gagarin 2001b).

#### Plectus rhizophilus

de Man, 1880

Plectus cirratus Bastian, 1865 var. rhizophilus de Man, 1880

##### Notes

Svalbard (Loof 1971, Zell 1993); Jan Mayen (Allgén 1953, Steiner 1916a); Greenland (Allgén 1954); Lena River estuary, Russia (Gagarin 2001b); Taymyr and Severnaya Zemlya, Russia (Gagarin and Kuzmin 1983, Kuzmin and Gagarin 1990); Novaya Zemlya and Vaigach island, Russia (Gagarin 1997a, Gagarin 2001b, Steiner 1916a).

#### Plectus rotundilabiatus

Zell, 1993

##### Notes

Iceland (Zell 1993).

#### Plectus tenuis

Bastian, 1865

##### Notes

Lena River estuary, Russia (Gagarin 2001b); Taymyr and Severnaya Zemlya, Russia (Gagarin 1996); Novaya Zemlya and Vaigach island, Russia (Gagarin 1997a, Gagarin 1999a, Gagarin 2001b).

#### Plectus varians

Maggenti, 1961

##### Notes

Iceland (Zell 1993); Taymyr and Severnaya Zemlya, Russia (Kuzmin 1976).

#### Plectus velox

Bastian, 1865

##### Notes

Svalbard (Zell 1993).

#### Tylocephalus auriculatus

(Bütschli, 1873)

Plectus auriculatus Bütschli, 1873

##### Notes

Jan Mayen (Steiner 1916a); Nunavut, Canada (Anderson 1966); Taymyr and Severnaya Zemlya, Russia (Kuzmin and Gagarin 1990).

#### Ereptonema arcticum

Loof, 1971*

##### Notes

Svalbard (Boström 1987a, Holovachov et al. 2003, Loof 1971).

#### Ereptonema fimbriatum

Anderson, 1966*

##### Notes

Nunavut, Canada (Anderson 1966).

#### Wilsonema otophorum

(de Man, 1880)

##### Notes

Taymyr and Severnaya Zemlya, Russia (Kuzmin and Gagarin 1990); Novaya Zemlya and Vaigach island, Russia (Gagarin 1997a, Gagarin 2001b).

#### Metateratocephalidae

#### Metateratocephalus crassidens

(de Man, 1880)

Teratocephalus crassidens de Man, 1880; *Euteratocephalus crassidens* (de Man, 1880)

##### Notes

Svalbard (Boström 1989, Loof 1971); Jan Mayen (Allgén 1953); Taymyr and Severnaya Zemlya, Russia (Kuzmin 1972, Kuzmin 1976, Kuzmin and Gagarin 1990); Novaya Zemlya and Vaigach island, Russia (Gagarin 1997a, Gagarin 2001b, Steiner 1916a).

#### Euteratocephalus palustris

(de Man, 1880)

##### Notes

Taymyr and Severnaya Zemlya, Russia (Kuzmin 1972, Kuzmin and Gagarin 1990).

#### Aulolaimidae

#### Aulolaimus oxycephalus

de Man, 1880

##### Notes

Taymyr and Severnaya Zemlya, Russia (Kuzmin 1986, Kuzmin and Gagarin 1990).

#### RHABDITIDA

#### Teratocephalidae

#### Teratocephalus costatus

Andrássy, 1958

Teratocephalus decarinus Anderson, 1969*

##### Notes

Svalbard (Boström 1989, Loof 1971); Nunavut, Canada (Anderson 1969); Taymyr and Severnaya Zemlya, Russia (Kuzmin and Gagarin 1990); Novaya Zemlya and Vaigach island, Russia (Gagarin 1997a, Gagarin 2001b).

#### Teratocephalus dadayi

Andrássy, 1968

Teratocephalus subvexus Anderson, 1969*

##### Notes

Nunavut, Canada (Anderson 1969).

#### Teratocephalus lirellus

Anderson, 1969*

##### Notes

Svalbard (Boström 1989, Loof 1971); Nunavut, Canada (Anderson 1969); Taymyr and Severnaya Zemlya, Russia (Kuzmin and Gagarin 1990).

#### Teratocephalus tenuis

Andrássy, 1958

##### Notes

Taymyr and Severnaya Zemlya, Russia (Kuzmin and Gagarin 1990).

#### Teratocephalus terrestris

(Bütschli, 1873)

##### Notes

Svalbard (Boström 1989); Jan Mayen (Allgén 1953); Taymyr and Severnaya Zemlya, Russia (Kuzmin 1972, Kuzmin 1976, Kuzmin and Gagarin 1990); Novaya Zemlya and Vaigach island, Russia (Steiner 1916b).

#### Alloionematidae

#### Rhabditophanes schneideri

(Bütschli, 1873)

Cheilobus quadrilabiatus Cobb, 1924

##### Notes

Taymyr and Severnaya Zemlya, Russia (Kuzmin 1972, Kuzmin and Gagarin 1990).

#### Panagrolaimidae

#### Panagrolaimus papillosus

Loof, 1971*

##### Notes

Svalbard (Loof 1971).

#### Panagrolaimus rigidus

(Schneider, 1866)

##### Notes

Svalbard (Loof 1971); Taymyr and Severnaya Zemlya, Russia (Kuzmin 1976, Kuzmin and Gagarin 1990).

#### Cephalobidae

#### Cephalobus filiformis

de Man, 1876

Cephalobus filiformis species inquirenda

##### Notes

Jan Mayen (Allgén 1953).

#### Cephalobus persegnis

Bastian, 1865

##### Notes

Svalbard (Loof 1971); Jan Mayen (Allgén 1953); Taymyr and Severnaya Zemlya, Russia (Kuzmin and Gagarin 1990).

#### Acrobeloides buetschlii

(de Man, 1884)

Cephalobus buetschlii de Man, 1884

##### Notes

Svalbard (Menzel 1920); Jan Mayen (Steiner 1916a); Taymyr and Severnaya Zemlya, Russia (Kuzmin and Gagarin 1990).

#### Acrobeloides enoploides

Loof, 1971*

##### Notes

Svalbard (Loof 1971).

#### Acrobeloides nanus

(de Man, 1880)

Cephalobus nanus de Man, 1880

##### Notes

Svalbard (Loof 1971).

#### Acrobeloides tricornis

(Thorne, 1925)

##### Notes

Svalbard (Boström 1987a, Loof 1971).

#### Eucephalobus arcticus

Loof, 1971*

##### Notes

Svalbard (Boström 1987a, Loof 1971).

#### Eucephalobus mucronatus

(Kozlovska & Roguska-Wasilewska, 1963)

##### Notes

Taymyr and Severnaya Zemlya, Russia (Kuzmin and Gagarin 1990).

#### Eucephalobus oxyuroides

(de Man, 1876)

Cephalobus oxyuroides de Man, 1876

##### Notes

Svalbard (Loof 1971); Jan Mayen (Allgén 1953).

#### Eucephalobus paracornutus

De Coninck, 1943

##### Notes

Taymyr and Severnaya Zemlya, Russia (Kuzmin 1972, Kuzmin and Gagarin 1990).

#### Eucephalobus striatus

(Bastian, 1865)

##### Notes

Taymyr and Severnaya Zemlya, Russia (Kuzmin 1972, Kuzmin and Gagarin 1990).

#### Pseudacrobeles elongatus

(de Man, 1880)

Cephalobus elongatus de Man, 1880; *Heterocephalobus elongatus* (de Man, 1880)

##### Notes

Svalbard (Loof 1971); Jan Mayen (Allgén 1953); Taymyr and Severnaya Zemlya, Russia (Gagarin 2001b, Kuzmin 1972, Kuzmin and Gagarin 1990).

#### Chiloplacus lentus

(Maupas, 1900)

##### Notes

Taymyr and Severnaya Zemlya, Russia (Kuzmin 1972, Kuzmin and Gagarin 1990).

#### Chiloplacus quintastriatus

Sumenkova in Sumenkova & Razjivin, 1968

Chiloplacus saccatus Loof, 1971*

##### Notes

Svalbard (Boström 1987a, Loof 1971).

#### Chiloplacus symmetricus

(Thorne, 1925)

##### Notes

Taymyr and Severnaya Zemlya, Russia (Kuzmin and Gagarin 1990).

#### Chiloplacus trilineatus

Steiner, 1940

##### Notes

Taymyr and Severnaya Zemlya, Russia (Kuzmin and Gagarin 1990).

#### Stegelletina insubrica

(Steiner, 1914)

Cervidellus insubricus (Steiner, 1914); *Ypsylonellus insubricus* (Steiner, 1914)

##### Notes

Taymyr and Severnaya Zemlya, Russia (Kuzmin 1972, Kuzmin and Gagarin 1990).

#### Stegelletina similis

(Thorne, 1925)

Stegelleta mucronata Loof, 1971*

##### Notes

Svalbard (Loof 1971).

#### Cervidellus vexilliger

(de Man, 1880)

Cervidellus serratus (Thorne, 1925); *Ypsylonellus vexilliger* (de Man, 1880)

##### Notes

Svalbard (Loof 1971); Taymyr and Severnaya Zemlya, Russia (Kuzmin 1972, Kuzmin and Gagarin 1990).

#### Cervidellus spitzbergensis

Boström, 1987*

##### Notes

Svalbard (Boström 1987b).

#### Acrobeles ciliatus

von Linstow, 1877

##### Notes

Svalbard (Loof 1971); Taymyr and Severnaya Zemlya, Russia (Kuzmin and Gagarin 1990).

#### Aphelenchoididae

#### Aphelenchoides arcticus

Sanwal, 1965*

##### Notes

Svalbard (Loof 1971); Nunavut, Canada (Sanwal 1965); Lena River estuary, Russia (Gagarin 2001b).

#### Aphelenchoides blastophthorus

Franklin, 1952

##### Notes

Lena River estuary, Russia (Gagarin 2001b).

#### Aphelenchoides echinocaudatus

Haque, 1968

##### Notes

Taymyr and Severnaya Zemlya, Russia (Kuzmin 1972, Kuzmin and Gagarin 1990).

#### Aphelenchoides goeldii

(Steiner, 1914)

Aphelenchus goeldii Steiner, 1914

##### Notes

Jan Mayen (Steiner 1916a).

#### Aphelenchoides parasaprophilus

Sanwal, 1965*

##### Notes

Nunavut, Canada (Sanwal 1965); Taymyr and Severnaya Zemlya, Russia (Gagarin 2001b).

#### Aphelenchoides parietinus

(Bastian, 1865)

Aphelenchus modestus de Man, 1876

##### Notes

Jan Mayen (Steiner 1916a); Taymyr and Severnaya Zemlya, Russia (Gagarin 1996, Kuzmin and Gagarin 1990); Novaya Zemlya and Vaigach island, Russia (Steiner 1916b).

#### Aphelenchoides robustus

Gagarin, 1997*

##### Notes

Novaya Zemlya and Vaigach island, Russia (Gagarin 1997c, Gagarin 1997a, Gagarin 2001b).

#### Aphelenchoides saprophilus

Franklin, 1957

##### Notes

Taymyr and Severnaya Zemlya, Russia (Kuzmin 1986, Kuzmin and Gagarin 1990).

#### Aphelenchoides subtenuis

(Cobb, 1926)

##### Notes

Taymyr and Severnaya Zemlya, Russia (Kuzmin 1972, Kuzmin and Gagarin 1990).

#### Tylenchidae

#### Aglenchus agricola

(de Man, 1884)

##### Notes

Greenland (Brzeski 1999); Taymyr and Severnaya Zemlya, Russia (Kuzmin and Gagarin 1990); Novaya Zemlya and Vaigach island, Russia (Gagarin 1997a, Gagarin 2001b).

#### Coslenchus costatus

(de Man, 1921)

Tylenchus costatus de Man, 1921

##### Notes

Svalbard (Loof 1971); Greenland (Brzeski 1999); Taymyr and Severnaya Zemlya, Russia (Kuzmin and Gagarin 1990); Novaya Zemlya and Vaigach island, Russia (Gagarin 1997a, Gagarin 2001b).

#### Basiria dolichura

Loof, 1971*

##### Notes

Svalbard (Loof 1971).

#### Lelenchus leptosoma

(de Man, 1880)

Tylenchus leptosoma de Man, 1880; *Anguillulina leptosoma* (de Man, 1880); *Filenchus leptosoma* (de Man, 1880)

##### Notes

Svalbard (Loof 1971); Jan Mayen (Allgén 1953); Taymyr and Severnaya Zemlya, Russia (Kuzmin 1986, Kuzmin and Gagarin 1990); Novaya Zemlya and Vaigach island, Russia (Steiner 1916b).

#### Filenchus cylindricaudus

(Wu, 1969)

Tylenchus cylindricaudus Wu, 1969*

##### Notes

Nunavut, Canada (Wu 1969b).

#### Filenchus cylindricus

(Thorne & Malek, 1968)

##### Notes

Lena River estuary, Russia (Gagarin 2001b).

#### Filenchus discrepans

(Andrássy, 1954)

Lelenchus discrepans Andrássy, 1954; *Ottolenchus discrepans* (Andrássy, 1954)

##### Notes

Taymyr and Severnaya Zemlya, Russia (Kuzmin 1986, Kuzmin and Gagarin 1990).

#### Filenchus ditissimus

(Brzeski, 1963)

Tylenchus ditissimus Brzeski, 1963

##### Notes

Taymyr and Severnaya Zemlya, Russia (Gagarin and Kuzmin 1983, Kuzmin and Gagarin 1990).

#### Filenchus facultativus

(Szczygiel, 1970)

##### Notes

Greenland (Brzeski 1999).

#### Filenchus filiformis

(Bütschli, 1873)

Filenchus filiformis species inquirenda; *Tylenchus filiformis* Bütschli, 1873; Tylenchus (Filenchus) filiformis Bütschli, 1873

##### Notes

Taymyr and Severnaya Zemlya, Russia (Kuzmin 1972, Kuzmin 1976, Kuzmin and Gagarin 1990); Novaya Zemlya and Vaigach island, Russia (Steiner 1916b).

#### Filenchus hamatus

(Thorne & Malek, 1968)

##### Notes

Greenland (Brzeski 1999).

#### Filenchus hazanensis

(Wu, 1969)

Tylenchus hazenensis Wu, 1969*; *Dactylotylenchus filiformis* Wu, 1969*

##### Notes

Nunavut, Canada (Wu 1969b, Wu 1969a).

#### Filenchus minutus

(Cobb, 1893)

Filenchus minutus species inquirenda

##### Notes

Taymyr and Severnaya Zemlya, Russia (Kuzmin and Gagarin 1990).

#### Filenchus misellus

(Andrássy, 1958)

##### Notes

Greenland (Brzeski 1999).

#### Filenchus orbus

Andrássy, 1954

Tylenchus aquilonius Wu, 1969*

##### Notes

Greenland (Brzeski 1999); Nunavut, Canada (Wu 1969b).

#### Filenchus quartus

(Szczygiel, 1969)

##### Notes

Greenland (Brzeski 1999).

#### Filenchus thornei

(Andrássy, 1954)

Tylenchus thornei Andrássy, 1954

##### Notes

Svalbard (Loof 1971); Taymyr and Severnaya Zemlya, Russia (Kuzmin and Gagarin 1990).

#### Malenchus acarayensis

Andrássy, 1968

##### Notes

Lena River estuary, Russia (Gagarin 2001b); Taymyr and Severnaya Zemlya, Russia (Kuzmin and Gagarin 1990).

#### Malenchus bryophilus

(Steiner, 1914)

Tylenchus bryophilus Steiner, 1914

##### Notes

Svalbard (Loof 1971); Novaya Zemlya and Vaigach island, Russia (Gagarin 1997a, Gagarin 2001b).

#### Malenchus exiguus

(Massey, 1969)

Ottolenchus sulcis Wu, 1970*

##### Notes

Nunavut, Canada (Wu 1970).

#### Tylenchus davainei

Bastian, 1865

Anguillulina davainei (Bastian, 1865); *Filenchus davainei* (Bastian, 1865)

##### Notes

Svalbard (Loof 1971); Jan Mayen (Allgén 1953); Greenland (Brzeski 1999, Ditlevsen 1927); Lena River estuary, Russia (Gagarin 2001b); Taymyr and Severnaya Zemlya, Russia (Gagarin and Kuzmin 1983, Kuzmin 1972, Kuzmin 1976, Kuzmin and Gagarin 1990); Novaya Zemlya and Vaigach island, Russia (Gagarin 1997a, Gagarin 2001b, Steiner 1916a).

#### Tylenchus neodavainei

Wu, 1969*

##### Notes

Nunavut, Canada (Wu 1969b).

#### Tylenchus stylolineatus

Wu, 1969*

##### Notes

Nunavut, Canada (Wu 1969b).

#### Dolichodoridae

#### Meiodorus hyalacus

(Anderson & Ebsary, 1982)

Mulveyotus hyalacus Anderson & Ebsary, 1982*

##### Notes

Nunavut, Canada (Anderson and Ebsary 1982).

#### Geocenamus arcticus

(Mulvey, 1969)

Tylenchorhynchus arcticus Mulvey, 1969*

##### Notes

Svalbard (Loof 1971); Nunavut, Canada (Mulvey 1969a); Taymyr and Severnaya Zemlya, Russia (Kuzmin 1986, Kuzmin and Gagarin 1990).

#### Geocenamus brevidens

(Allen, 1955)

Merlinius brevidens (Allen, 1955)

##### Notes

Taymyr and Severnaya Zemlya, Russia (Kuzmin and Gagarin 1990).

#### Geocenamus circellus

(Anderson & Ebsary, 1982)

Merlinius circellus Anderson & Ebsary, 1982*

##### Notes

Nunavut, Canada (Anderson and Ebsary 1982).

#### Geocenamus joctus

(Thorne, 1949)

Tetylenchus joctus Thorne, 1949

##### Notes

Taymyr and Severnaya Zemlya, Russia (Kuzmin 1986, Kuzmin and Gagarin 1990).

#### Geocenamus loofi

(Siddiqi, 1979)

Tetylenchus joctus sensu Loof, 1971; *Merlinius loofi* Siddiqi, 1979

##### Notes

Svalbard (Loof 1971, Siddiqi 1979).

#### Geocenamus microdorus

(Geraert, 1966)

Tylenchorhynchus microdorus Geraert, 1966

##### Notes

Svalbard (Loof 1971).

#### Geocenamus tetylus

(Anderson & Ebsary, 1982)

Merlinius tetylus Anderson & Ebsary, 1982*

##### Notes

Nunavut, Canada (Anderson and Ebsary 1982).

#### Nagellus borealis

Powers, Baldwin & Bell, 1983*

##### Notes

Alaska (Powers et al. 1983).

#### Nagellus grandis

(Allen, 1955)

Tylenchorhynchus grandis Allen, 1955

##### Notes

Taymyr and Severnaya Zemlya, Russia (Kuzmin 1972, Kuzmin and Gagarin 1990).

#### Nagelus leptus

(Allen, 1955)

Tylenchorhynchus leptus Allen, 1955

##### Notes

Svalbard (Loof 1971); Greenland (Brzeski 1999); Nunavut, Canada (Mulvey 1969a); Taymyr and Severnaya Zemlya, Russia (Kuzmin and Gagarin 1990).

#### Nagellus lineatus

(Allen, 1955)

##### Notes

Taymyr and Severnaya Zemlya, Russia (Kuzmin and Gagarin 1990).

#### Nagellus obscurus

(Allen, 1955)

##### Notes

Taymyr and Severnaya Zemlya, Russia (Kuzmin and Gagarin 1990).

#### Nagellus parobscurus

(Mulvey, 1969)

Tylenchorhynchus parobscurus Mulvey, 1969*

##### Notes

Nunavut, Canada (Mulvey 1969a).

#### Tylenchorhynchus dubius

(Bütschli, 1873)

Bitylenchus dubius (Bütschli, 1873)

##### Notes

Taymyr and Severnaya Zemlya, Russia (Kuzmin 1972, Kuzmin 1976, Kuzmin and Gagarin 1990).

#### Tylenchorhynchus nanus

Allen, 1955

Geocenamus nanus (Allen, 1955); *Merlinius nanus* (Allen, 1955)

##### Notes

Greenland (Brzeski 1999); Taymyr and Severnaya Zemlya, Russia (Kuzmin 1986, Kuzmin and Gagarin 1990).

#### Tylenchorhynchus cf. nothus

Allen, 1955

##### Notes

Svalbard (Loof 1971).

#### Tylenchorhynchus parvus

Allen, 1955

Bitylenchus parvus (Allen, 1955)

##### Notes

Taymyr and Severnaya Zemlya, Russia (Kuzmin and Gagarin 1990).

#### Hoplolaimidae

#### Rotylenchus brevicaudatus

(Hopper, 1959)

Pararotylenchus brevicaudatus (Hopper, 1959)

##### Notes

Taymyr and Severnaya Zemlya, Russia (Kuzmin and Gagarin 1990).

#### Helicotylenchus platyurus

Perry in Perry, Darling & Thorne, 1959

##### Notes

Greenland (Brzeski 1999).

#### Helicotylenchus spitsbergensis

Loof, 1971*

##### Notes

Svalbard (Loof 1971); Greenland (Brzeski 1999).

#### Helicotylenchus varicaudatus

Yuen, 1964

##### Notes

Taymyr and Severnaya Zemlya, Russia (Kuzmin 1972, Kuzmin and Gagarin 1990).

#### Pratylenchidae

#### Pratylenchoides crenicauda

Winslow, 1958

##### Notes

Svalbard (Loof 1971).

#### Pratylenchoides magnicauda

(Thorne, 1935)

Tylenchorhynchus magnicauda (Thorne, 1935)

##### Notes

Svalbard (Loof 1971).

#### Pratylenchus pratensis

(de Man, 1880)

Anguillulina pratense (de Man, 1880)

##### Notes

Jan Mayen (Allgén 1953).

#### Hirschmanniella gracilis

(de Man, 1880)

Tylenchus gracilis Cobb (lapsus)

##### Notes

Svalbard (von Linstow 1900).

#### Hirschmanniella trimucronata

Gagarin, 2000*

##### Notes

Novaya Zemlya and Vaigach island, Russia (Gagarin 1997a, Gagarin 2000, Gagarin 2001b).

#### Criconematidae

#### Criconemoides annulatus

Cobb in Taylor, 1936

Criconemoides hemispaericaudatus Wu, 1965*

##### Notes

Svalbard (Loof 1971); Nunavut, Canada (Wu 1965).

#### Tylenchulidae

#### Paratylenchus macrophallus

(de Man, 1880)

Paratylenchus macrophallus species inquirenda; *Anguillulina macrophallum* (de Man, 1880)

##### Notes

Jan Mayen (Allgén 1953).

#### Paratylenchus cf. projectus

Jenkins, 1956

##### Notes

Greenland (Brzeski 1999).

#### Anquinidae

#### Anguina agrostis

(Steinbuch, 1799)

##### Notes

Nunavut, Canada (Mulvey 1963).

#### Subanguina askenasyi

(Bütschli, 1873)

Ditylenchus askenasyi (Bütschli, 1873)

##### Notes

Taymyr and Severnaya Zemlya, Russia (Gagarin 2001b).

#### Ditylenchus acutatus

Brzeski, 1991

##### Notes

Greenland (Brzeski 1999).

#### Ditylenchus dryadis

Anderson & Mulvey, 1980*

##### Notes

Nunavut, Canada (Anderson and Mulvey 1980).

#### Ditylenchus equalis

Heyns, 1964

##### Notes

Greenland (Brzeski 1999).

#### Ditylenchus intermedius

(de Man, 1880)

##### Notes

Taymyr and Severnaya Zemlya, Russia (Kuzmin 1972, Kuzmin and Gagarin 1990).

#### Nothotylenchus acris

Thorne, 1941

##### Notes

Nunavut, Canada (Mulvey 1969b); Taymyr and Severnaya Zemlya, Russia (Kuzmin and Gagarin 1990).

#### Nothotylenchus acutus

Khan, 1965

##### Notes

Nunavut, Canada (Mulvey 1969b).

#### Nothotylenchus attenuatus

Mulvey, 1969*

##### Notes

Nunavut, Canada (Mulvey 1969b).

#### Nothotylenchus cf. danubialis

Andrássy, 1960

##### Notes

Nunavut, Canada (Mulvey 1969b).

#### Neotylenchidae

#### Deladenus durus

(Cobb, 1922)

##### Notes

Nunavut, Canada (Das 1964).

#### Hadrodenus megacondylus

Mulvey, 1969*

##### Notes

Nunavut, Canada (Mulvey 1969b).

#### Hexatylus mulveyi

Das, 1964*

##### Notes

Nunavut, Canada (Das 1964).

#### Prothallonema mucronatum

(Thorne & Malek, 1968)

Stictylus mucronatus Thorne & Malek, 1968

##### Notes

Greenland (Brzeski 1999); Nunavut, Canada (Mulvey 1969b).

#### Bunonematidae

#### Bunonema hessi

Steiner, 1914

##### Notes

Novaya Zemlya and Vaigach island, Russia (Steiner 1916b).

#### Bunonema reticulatum

Richters, 1905

##### Notes

Svalbard (Loof 1971).

#### Diplogastridae

#### Diplogaster rivalis

(Leydig, 1854)

##### Notes

Lena River estuary, Russia (Gagarin 2001b).

#### Goffartia variabilis

(Micoletzky, 1922)

##### Notes

Taymyr and Severnaya Zemlya, Russia (Gagarin 1990, Gagarin 2001b, Gagarin 2001a, Gagarin 2008).

#### Fictor fictor

(Bastian, 1865)

##### Notes

Taymyr and Severnaya Zemlya, Russia (Kuzmin and Gagarin 1990).

#### Fictor shoshini

Gagarin, 1995*

##### Notes

Lena River estuary, Russia (Gagarin 1995, Gagarin 2001b); Novaya Zemlya and Vaigach island, Russia (Gagarin 1997a).

#### Koerneria angarensis

(Gagarin, 1983)

Fictor angarensis (Gagarin, 1983)

##### Notes

Lena River estuary, Russia (Gagarin 2001b); Taymyr and Severnaya Zemlya, Russia (Gagarin 1990, Gagarin 2001b, Gagarin 2001a).

#### Koerneria ruricola

(Gagarin, 1983)

Fictor ruricola (Gagarin, 1983)

##### Notes

Taymyr and Severnaya Zemlya, Russia (Gagarin 2001b).

#### Mononchoides intermedius

Gagarin, 1993

##### Notes

Taymyr and Severnaya Zemlya, Russia (Gagarin 1996, Gagarin 2001b).

#### Rhabditidae

#### Choriorhabditis gracilicauda

(de Man, 1876)

Rhabditis gracilicauda de Man, 1876

##### Notes

Jan Mayen (Allgén 1953).

#### Choriorhabditis producta

(Schneider, 1866)

Anguillulina intermedia (de Man, 1880)

##### Notes

Jan Mayen (Allgén 1953).

#### Rhabditis terricola

Dujardin, 1844

Rhabditis aspera Bütschli, 1873

##### Notes

Jan Mayen (Allgén 1953).

#### Aphelenchus nivalis

Aurivillius, 1883*

##### Notes

Inquirenda et incertae sedis. Svalbard (Aurivillius 1883).

#### Macfadyenia filicaudata

Allgén, 1953*

##### Notes

Inquirenda et incertae sedis. Jan Mayen (Allgén 1953).

#### Parachromagasteriella arctica

Allgén, 1953*

##### Notes

Inquirenda et incertae sedis. Jan Mayen (Allgén 1953).

#### Plectus cirratusf.parietinus sf. latilaimus

Allgén, 1953*

##### Notes

Inquirenda et incertae sedis. Jan Mayen (Allgén 1953).

#### Prodomorganus tajmiris

Gagarin, 1993*

##### Notes

Inquirenda et incertae sedis. Taymyr and Severnaya Zemlya, Russia (Gagarin 1990, Gagarin 1993, Gagarin 2001b).

#### Teratocephalus micrurus

Rahm, 1924

##### Notes

Inquirenda et incertae sedis. Taymyr and Severnaya Zemlya, Russia (Kuzmin 1972, Kuzmin and Gagarin 1990).

#### Tylenchus macrospiculatus

Gagarin, 1997

##### Notes

Nomen nudum. Novaya Zemlia and Vaigach island, Russia (Gagarin 1997a).

## Discussion

Despite of more than 130 years of research, our knowledge on the diversity and distribution of Arctic nematodes is still very incomplete and limited to only few relatively small areas of the region. The first studies of Arctic nematodes date back to 1883, when the first nematode was described from Spitzbergen (Aurivillius 1883). The taxonomic position of the species *Aphelenchus nivalis* Aurivillius, 1883 remains uncertain and its type material can not be located for re-study. After Aurivillius, the nematode fauna of Svalbard archipelago was studied by von Linstow (1900), Menzel (1920), Boström (1987a), Boström (1987b), Boström (1989), Zell (1993) and to a lesser extent by Brzeski (1962), Siddiqi (1979), Janiec (1997), Klekowski and Opalinski (1986), Klekowski and Opalinski (1990), Klekowski and Opalinski (1992), Opalinski and Klekowski (1992), Holovachov et al. (2003) adding up to the list of 101 species. Most of the sampling on Svalbard was concentrated around the west part of the Spitsbergen island and a large part of the archipelago remains unstudied. From other territories belonging to the Kingdom of Norway, 43 species of nematodes were found on the small island of Jan Mayen (Allgén 1953, Steiner 1916a), while no information is available for the northernmost part of the Finnmark.

Most of the nematological research in the Iceland was focused in southern and western part of the island (De Coninck 1940, De Coninck 1943, De Coninck 1944), which is considered as Subarctic in the current publication. Sampling sites from the northern part of the country were also outside the boundary considered in the present paper (Kreis 1963, Starmühlner 1969). A small part of the Arctic of the Iceland has not been studied to any considerable extent – only six species from the genus *Plectus* are listed from the areas near Húsavík and Tjörnes by Zell (1993). Terrestrial nematode fauna of Greenland, on the other hand, received considerable attention from Ditlevsen (1927), Allgén (1954), Zell (1993), Brzeski (1999), who altogether reported 43 species of nematodes collected in several distant localities along the eastern (Little Pendulum Island and Angmagssalik) and western (Disko island, Kangerlussuaq, Kapisillit, Sukkertoppen and Upernavik) sides of the island.

Nematode fauna of the Arctic regions of Canada is studied comparatively better. The research was focused in several localities in the Nunavut province including Bathhurst, Ellef Ringnes, Ellesmere and Somerset islands (Anderson 1966, Anderson 1969, Anderson and Ebsary 1982, Anderson and Mulvey 1980, Das 1964, Ebsary 1984, Ebsary 1985, Elshishka et al. 2013, Mulvey 1963, Mulvey 1967b, Mulvey 1967a, Mulvey 1969c, Mulvey 1969a, Mulvey 1969b, Mulvey 1978, Sanwal 1965, Sanwal 1968, Wu 1965, Wu 1969b, Wu 1969a, Wu 1970, Zell 1993), and in the small area of the Mackenzie River delta and Tuktoyaktok in the Northwest territories (Ebsary 1982, Eveleigh 1982, Mulvey 1978, Mulvey and Anderson 1979). Respectively, 55 and 13 species were described from these regions between 1963 and 1993. Much less is known about the nematodes from the Arctic regions of Alaska – only 22 species were recorded there by Andrássy (2003a), Andrássy (2003b), Andrássy (2003c), Bernard (1992), Powers et al. (1983). Studied localities include Prudhoie Bay and neighbouring North Slope and Brooks Range, as well as localities along Kobuk River near Noorvik and Unalakleet River near Unalakleet.

Fauna of the Russian part of the Arctic lists the largest number of nematode species, including many typical freshwater nematodes, but known from only three distinct geographic regions: Novaya Zemlya archipelago together with Vaygach island, Taymyr peninsula together with Severnaya Zemlya, and the Lena River estuary. So far, only 37 species of freshwater nematodes are know from the Lena River estuary (Gagarin 1995, Gagarin 1999b, Gagarin 2000, Gagarin 2001b). The list of nematodes from the Novaya Zemlya archipelago and Vaygach island includes 81 nematode species, both terrestrial (Steiner 1916b) and freshwater (Gagarin 1997c, Gagarin 1997b, Gagarin 1997a, Gagarin 1999a, Gagarin 2000, Gagarin 2001c, Gagarin 2001b). Arctic areas of the Taymyr peninsula and Severnaya Zemlya are the most intensively studied part of the Arctic with localities including the Taymyr Lake, Enisei River, Putorana Plateau, Cape Chelyuskin – 229 species of freshwater and terrestrial nematodes were reported from different sampling sites of this region over the past 40 years (Elshishka et al. 2012, Elshishka et al. 2013, Gagarin 1990, Gagarin 1991a, Gagarin 1991b, Gagarin 1993, Gagarin 1996, Gagarin 1999a, Gagarin 2001b, Gagarin 2001a, Gagarin 2008, Gagarin 2009, Gagarin and Kuzmin 1972, Gagarin and Kuzmin 1983, Kuzmin 1972, Kuzmin 1976, Kuzmin 1986, Kuzmin and Gagarin 1990).

In summary, 391 species, belonging to 146 genera, 54 families, and 10 orders of the phylum Nematoda are known from the entire Arctic (including species of dubious taxonomic status, but exclusing single nomen nudum). Even taking into consideration relatively low variety of habitats in this region, comparing to other areas of the world, as well as the rather harsh environmental conditions, this list is considered to be very incomplete and preliminary.
