# Corrigendum: Nematodes from terrestrial and freshwater habitats in the Arctic

**DOI:** 10.3897/BDJ.4.e7768

**Published:** 2016-01-14

**Authors:** Oleksandr Holovachov

**Affiliations:** ‡ Swedish Museum of Natural History, Stockholm, Sweden

## Note

In preparation of the manuscript (Holovachov 2014) the author missed to include one important reference in the first paragraph of the discussion. The updated version of the relevant sentence is given below with missed text marked in bold.

"After Aurivillius, the nematode fauna of Svalbard archipelago was studied by von Linstov (1900), Menzel (1920), **Loof (1971)**, Boström (1987a), Boström (1987b), Boström (1989), Zell (1993) and to a lesser extent by Brzeski (1962), Siddiqi (1979), Janiec (1977), Klekowski and Opalinski (1986), Klekowski and Opalinski (1990), Klekowski and Opalinski (1992), Opalinski and Klekowski (1992), Holovachov et al. (2003) adding up to the list of 101 species."
